# Assessment of PlanetScope Spectral Data for Estimation of Peanut Leaf Area Index Using Machine Learning and Statistical Methods

**DOI:** 10.3390/s26031018

**Published:** 2026-02-04

**Authors:** Michael Ekwe, Hansanee Fernando, Godstime James, Oluseun Adeluyi, Jochem Verrelst, Angela Kross

**Affiliations:** 1 Department of Strategic Space Application, National Space Research and Development Agency, Airport Road, P.M.B. 437, Abuja 900101, Nigeria; godstimej@gmail.com (G.J.); seun.adeluyi@nasrda.gov.ng (O.A.); 2 Department of Geography, Planning and Environment, Concordia University, Montréal, QC H3G 1M8, Canada; angela.kross@concordia.ca; 3 Department of Plant Sciences, College of Agriculture and Bioresources, University of Saskatchewan, Saskatoon, SK S7N 5A8, Canada; hansanee.fernando@usask.ca; 4 Image Processing Laboratory (IPL), Parc Científic, Universitat de València, 46980 Paterna, Spain; jochem.verrelst@uv.es

**Keywords:** LAI, PlanetScope, spectral bands, vegetation indices, machine learning models

## Abstract

Leaf area index (LAI) is a key indicator of crop growth and development and is widely used in both agricultural research and precision farming applications. PlanetScope imagery is generally used for monitoring crop growth due to its high revisit frequency, broad spatial coverage, and cost-effective access to consistent high-resolution multispectral data. Therefore, we developed regression models to estimate peanut LAI, combining PlanetScope spectral bands and vegetation indices (VIs). Specifically, we compared the performance of random forest (RF), eXtreme Gradient Boosting (XGBoost), and Partial Least Squares Regression (PLSR) regression algorithms for peanut LAI estimation. Our results showed that most of the VIs exhibited strong relationships with LAI. Thirteen VIs were individually evaluated for estimating LAI using the aforementioned algorithms, and our results showed that the best single predictors of LAI are: TSAVI (RF: R^2^ = 0.87, RMSE = 0.83 m^2^/m^2^, RRMSE = 24.20%; XGBoost: R^2^ = 0.77, RMSE = 0.95 m^2^/m^2^, RRMSE = 27.96%); and RTVIcore (PLSR: R^2^ = 0.68, RMSE = 1.12 m^2^/m^2^, RRMSE = 32.88%). The top six ranked VIs were used to calibrate the RF, XGBoost, and PLSR algorithms. Model validation indicated that RF achieved the highest accuracy (R^2^ = 0.844, RMSE = 0.858 m^2^/m^2^, RRMSE = 25.17%), followed by XGBoost (R^2^ = 0.808, RMSE = 0.92 m^2^/m^2^, RRMSE = 26.99%), whereas PLSR showed comparatively lower performance (R^2^ = 0.76, RMSE = 0.983 m^2^/m^2^, RRMSE = 28.85%). Further results showed that PlanetScope VIs provided superior model accuracy in estimating peanut LAI compared to the use of spectral bands alone. Additionally, integrating spectral bands with VIs reduced LAI estimation accuracy, underscoring the importance of selecting predictor variables in ensuring optimal model performance. Overall, the presented results are significant for future crop monitoring using RF to reduce overreliance on multiple models for peanut LAI estimation.

## 1. Introduction

Projected human population growth necessitates optimized crop yields to ensure food security amidst increasing climate-driven extreme weather events. Monitoring crop development with high-resolution satellite Earth observation data supports informed, timely agricultural decisions such as precise timing of fertilizer applications [1], irrigation scheduling [2], tracking diseases and pests [3], weed control [4], and crop yield estimation [5]. Leaf area index (LAI), defined as one half the total green leaf area per unit ground surface area [6], serves as a key indicator of monitoring overall plant health and estimating yield [7], and can be used indirectly as an input parameter in ecosystem productivity and crop simulation models [8]. Traditionally, LAI is estimated by using either direct or indirect methods. Direct methods rely on destructive sampling and are highly accurate but labor-intensive, whereas indirect methods employ allometric relationships or optical proximal sensors to estimate LAI. The increasing availability of high-tech sensors and computational capability of smartphone devices have paved the way for the development of low-cost apps for indirect LAI monitoring [9]. Recently, the VitiCanopy app was developed to accurately estimate the canopy vigor and porosity in vegetation canopies [10]. Previous studies have shown that LAI estimates from VitiCanopy app demonstrated good agreement with estimates measured by the LAI-2000 Plant Canopy Analyzer (Applied Biosystems, Waltham, MA, USA) [10]. However, despite its low cost and practicality, the application of this approach for peanut LAI monitoring remains largely unexplored in crop monitoring studies, with only limited evidence available in the literature [11].

Satellite Earth observation data has been primarily used in previous studies for LAI estimation due to its time, cost, and labor savings for large crop areas. Predominantly, vegetation indices (VIs) derived from optical remote sensing data have been widely used to estimate crop LAI [12,13]. Overall, distinct VIs exhibit varying sensitivity to LAI estimates. For example, the traditional normalized difference vegetation index (NDVI) has shown a strong relationship with LAI; however, it saturates at medium to high LAI values [12,13]. Simple ratio (SR), modified triangular vegetation index 2 (MTVI2), and cumulative MTVI2 have shown improved sensitivity at medium to high LAI values [13,14]. Similarly, VIs that incorporate red-edge reflectance bands, such as the Red-Edge Triangular Vegetation Index (RTVI) and the Modified Chlorophyll Absorption Ratio Index (MCARI2), have shown enhanced potential for accurate LAI estimation at medium to high values [14]. Du et al. [15] showed that RGB-based VIs generated from UAV imagery were strongly correlated with maize LAI. Li et al. [16] established that the visible atmospherically resistant index (VARI) was the best for rice LAI estimation.

Accurate crop monitoring in precision agriculture requires high spatio-temporal resolution Earth observation data, which PlanetScope provides through near-daily 3 m imagery with a red-edge band, and PlanetScope-derived vegetation indices have been successfully used for yield and LAI estimation across several crops [17,18]. While peanut LAI has been assessed using UAV and Sentinel-2 data [11,19], its estimation using PlanetScope imagery remains largely unexplored, representing a clear gap that this study aims to address. This study addresses this gap by evaluating the capability of PlanetScope data for monitoring peanut LAI, and by identifying the key spectral bands and vegetation indices that drive model performance. To translate these spectral features into accurate LAI estimates, advanced nonparametric machine learning (ML) and statistical approaches are applied to model the complex and nonlinear relationships between PlanetScope data and canopy structure. ML and nonparametric statistical algorithms are increasingly applied in remote sensing to estimate crop biophysical parameters such as LAI and biomass, with linear approaches like PLSR showing reasonable performance but limited capability in capturing complex nonlinear relationships [20]. Comparative studies have demonstrated that nonlinear ML methods (e.g., RF, SVR, XGBoost) generally outperform parametric models, particularly under limited sample sizes and noisy conditions, making them well suited for robust LAI and biomass retrieval from Earth observation data [21,22].

Therefore, the specific objectives of this study are to: (1) investigate the relationships between LAI and PlanetScope-derived VIs, (2) identify the important spectral features (bands or VIs) with the highest predictive power for estimating peanut LAI, and (3) evaluate and compare the estimation performance of RF, XGBoost, and PLSR algorithms in estimating peanut LAI using PlanetScope imagery. Together, these objectives address the lack of dedicated evaluations of PlanetScope for peanut LAI and the limited understanding of which spectral features and modeling approaches are most effective for this crop, thereby providing a more reliable and interpretable framework for operational LAI monitoring in peanut production systems.

## 2. Data and Methods

### 2.1. Study Area Description

**Figure 1:**
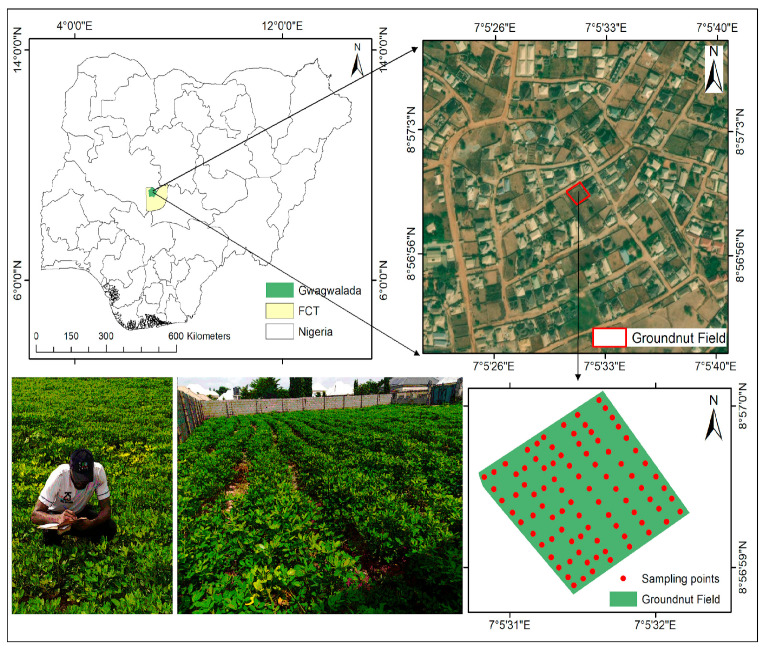
Geographical location and layout of the peanut experimental site, showing LAI sampling points (red dots) collected on 22 May 2022, 33 days after planting at the BBCH 33/34 (stem elongation) growth stage (Figure 1 was adapted from Ekwe et al. [11]).

The smallholder farmer’s experimental study site is located in the tropical north-central region of the Federal Capital Territory (FCT), Abuja, Nigeria. The site covers an area of 3712.45 m^2^ and is situated at the coordinates: latitude (8°56′58.92″ N~8°57′0.054″ N) and longitude (7°5′30.804″ E~7°5′32.172″ E) (Figure 1). The study area is largely covered by fertile alluvial soils that are clayey, loamy, and silty, providing ideal conditions for peanut crop cultivation. According to the Köppen climate classification system, the region experiences a tropical wet and dry climate (*Aw*). The weather in the region is primarily influenced by orographic features and the dynamic interaction between two dominant air masses: the tropical continental (*cT*) air mass and the tropical maritime (*mT*) air mass [23]. The region witnesses the rainy season between mid-March and October, which is important for peanut production. During the dry season around March, daytime temperatures in the region can reach up to 39 °C, whereas during the Harmattan period (December–January), nighttime temperatures can drop to as low as 17 °C [11]. The optimal soil temperature for effective germination and vegetative growth of peanut ranges between 27 °C and 30 °C, while the ideal temperature for the reproductive growth phase lies between 24 °C and 27 °C; additionally, annual rainfall between 450 mm and 1250 mm is required for optimum growth and yield [24]. The experimental site is well-known for its crop rotational cultivation of peanut, corn (*Zea mays*), and Bambara groundnuts (*Vigna subterranea*).

### 2.2. Experimental Design and In Situ Data Measurement

In the experimental field, peanut was manually cultivated under rainfed conditions using the medium-maturing variety Samnut-22, immediately after the onset of the rainy season on 19 April 2022. Seeding was conducted along clearly defined ridges within the field, which covered a total area of 3712.45 m^2^. A total of 89 sampling points were randomly selected for in situ LAI measurements, and their geographic coordinates were recorded using a Garmin eTrex 10 GPS device (Garmin Ltd., Schaffhausen, Switzerland); sampling points were spaced at least 3 m apart to avoid multiple points within a single image pixel. After data cleaning and outlier removal, 83 LAI measurements were retained for subsequent analysis in this study. LAI measurement was conducted using the Viticanopy Smart-App installed on Tecno Spark 5 Pro (Tecno Mobile, Transsion Holdings, Shenzhen, China) with Android operating system. Before data collection, the gap fraction was set to the default 75%, following De Bei et al. [10]. VitiCanopy utilizes the smartphone’s front camera and GPS to capture upward-looking canopy images and automatically calculate parameters such as LAI, canopy cover, and porosity. Further details regarding the description and configuration settings of the app can be found in De Bei et al. [10]. The Viticanopy Smart-App has been successfully tested and validated in previous studies for monitoring LAI and phenology in different crop and vegetation types, including vineyards [25], orchards [26], and groundnut [11].

### 2.3. Satellite Data

#### PlanetScope Imagery and Preprocessing

Cloud-free PlanetScope level-3 surface reflectance imagery collected on 22 May 2022 was downloaded from the Planet Explorer website (https://www.planet.com/explorer/; accessed on 25 October 2024). In this study, the PSB.SD sensor, Dove CubeSat from the PS SuperDove series, was utilized. The PSB.SD instrument offers eight spectral bands, namely red-edge, red, green, green I, yellow, blue, coastal blue, and near-infrared (NIR), with a spatial resolution of 3 m and a near-daily global revisit frequency. The PlanetScope orthorectified product was processed for geometric and radiometric corrections to account for Bottom-Of-Atmosphere (BOA) surface reflectance and projected into the UTM/WGS84 coordinate reference system [27].

### 2.4. Remote Sensing Variables

We used a total of thirteen VIs and five PlanetScope surface reflectance bands to estimate peanut LAI (Table 1). All the VIs were generated using the ‘*Indices*’ tool available within the ArcGIS Pro software environment (ESRI, Redlands Inc., Redlands, CA, USA). We used the coordinates of each sampling location and retrieved all the matching VIs and bands using the ‘*Extract Multi Values to Points*’ function within the Spatial Analyst tools in ArcGIS Pro software.

### 2.5. Modeling Methods

Two ML (RF and XGBoost) and one linear nonparametric (PLSR) models were chosen to guarantee robust and accurate LAI estimations using different algorithms. These models were selected based on their advantages and relevance for estimating LAI using PlanetScope-derived spectral reflectance bands and VIs. The selection of these algorithms enables a comprehensive evaluation of model performance and the identification of the most effective approach for estimating peanut LAI.

RF is a nonparametric ensemble learning method derived from the principles of CART (classification and regression trees), and it encompasses various trees that are trained using a bagging and random variable selection approach [39]. The RF algorithm is robust to outliers and noise [40], making it well-suited for handling overfitting and complex datasets. Previous studies have demonstrated its accuracy in estimating vegetation biophysical parameters such as LAI [41]. In this study, the Variable Selection Using Random Forests (VSURF) was used to determine the most important predictor variables for estimating the peanut LAI. RF modeling was performed using the “*randomForest*” package in base R. In the RF model, default hyperparameters were applied, with the number of trees (*ntree*) set to 500 and the number of features considered at each split (*mtry*) set to 3. Several studies have identified 500 trees as an optimal number for the RF model, as increasing the number of trees beyond this threshold does not lead to significant improvements in estimation accuracy [42]. We deployed the built-in *importance()* function in the ‘*randomForest*’ package to provide important predictor variables using the Percent Increase in Mean Squared Error (%IncMSE) metric [39,43]. The higher a variable’s %IncMSE value is, the more important the predictor variable is [44].

XGBoost is a powerful ensemble learning model that integrates Gradient Boosting with advanced regularization approaches to improve estimation performance [45]. It employs an iterative learning process in which errors from previous iterations are incorporated to enhance the performance of subsequent iterations, thereby improving overall model accuracy. Through regularization, the XGBoost algorithm reduces overfitting, handles missing data, and requires less training and estimation time, making it highly robust for large-scale data [46]. The XGBoost model was chosen for its exceptional performance in crop LAI mapping, as evidenced by previous research [47,48,49]. In this study, the model was fine-tuned using different hyperparameters, including the number of boosting rounds (*nrounds* = 500), learning rate (*eta* = 0.1), maximum depth of each decision tree (*max_depth* = 6), and the evaluation metric (*eval_metric*) was set to root mean square error. XGBoost modeling was performed using the “*xgboost*” package in base R. We used the built-in *xgb.importance()* function in the *xgboost* package to provide important predictor variables using the Gain% feature importance metric.

PLSR is a widely applied multivariate statistical technique commonly employed for developing estimation models, particularly in scenarios where the explanatory variables are numerous, highly correlated, and exhibit multicollinearity [50]. It reduces dimensionality and noise by transforming collinear input features into a smaller set of uncorrelated latent variables through component projection; however, interpreting the coefficients can be challenging [51]. Prior research has shown PLSR to be one of the effective nonparametric linear methods for estimating LAI [52,53,54]. In this study, PLSR modeling was performed using the “*pls*” package in base R with five PLS components, and the model was fitted with the kernel algorithm. For PLSR modeling, the *Variable importance in projection (VIP)* function was used to select the important features [55].

These RF, XGBoost, and PLSR algorithms provide robust and reliable estimation of crop biophysical parameters such as LAI, with their respective advantages and limitations summarized in Table 2.

### 2.6. Statistical Evaluation

Data partitioning was conducted using the “*createdatapartition()*” function from the caret package in base R, allocating 70% of the data for training and 30% for validation. We assessed the relationship between LAI and spectral features using linear regression analysis, quantified as the coefficient of determination (R^2^). The assessment of model performance was performed by adopting the root mean square error (RMSE), relative RMSE (RRMSE%), and coefficient of determination (R^2^). The equations of these evaluation metrics were expressed as follows:

(1) $$R^{2}=1-\frac{\sum_{k=1}^{n}{\left(y_{k}-\hat{y_{k}}\right)}^{2}}{\sum_{k=1}^{n}{\left(y_{k}-\bar{\hat{y_{k}}}\right)}^{2}}$$

(2) $$RMSE=\sqrt{\frac{1}{n}\sum_{k=1}^{n}{\left(y_{k}-\hat{y_{k}}\right)}^{2}}$$

(3) $$RRMSE=\frac{RMSE}{\bar{y_{k}}}\times 100$$

where $y_{k}$ is the measured LAI, $\hat{y_{k}}$ is the estimated LAI, $\bar{y_{k}}$ and $\bar{\hat{y_{k}}}$ are the average measured and estimated LAI, respectively, and n is the number of observations used for model validation.

### 2.7. Generation of LAI Estimation Maps

Inverse distance weighted (IDW) interpolation was used to map the spatial variability of the measured LAI and compare it with model-based LAI estimates. To create the peanut LAI estimation maps, raster layers of the most important VIs were needed. The raster layers of the VIs were generated using the “indices” tools in ArcGIS Pro software (ESRI, Redlands Inc., Redlands, CA, USA). The top six VIs, identified based on their feature importance for the RF, XGBoost, and PLSR algorithms respectively, were selected to generate the final LAI estimation maps. In the base R, “*library(raster)*”, “*library(rgdal)*”, and “*library(rasterVis)*” were utilized to create the final LAI estimation maps. The input raster layers (i.e., the VIs) were stacked using the “*stack ()*” function, and the “*raster::predict ()*” function was deployed to estimate LAI using the stacked layers and each algorithm. The outputs were exported as Geotiff files into the ArcGIS Pro software to create the final LAI estimation maps.

## 3. Results and Discussion

### 3.1. The Descriptive Statistics of Peanut LAI Measurements

**Figure 2:**
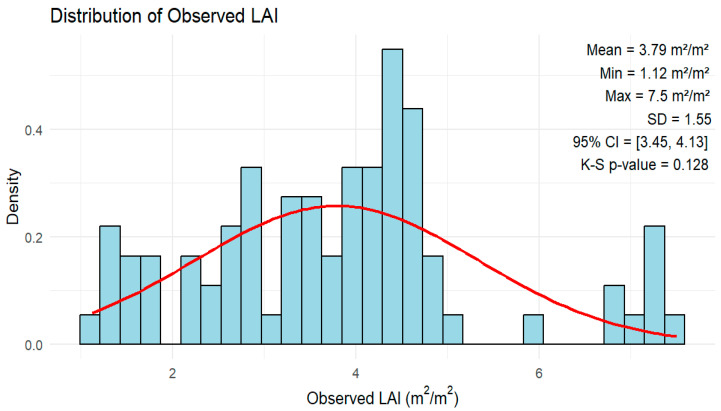
Descriptive statistics of observed peanut LAI. The red line represents the normal distribution fitted using the observed LAI mean and standard deviation.

The normality of the LAI data was assessed using the Kolmogorov–Smirnov (K–S) test with a *p*-value of 0.128 (Figure 2). The distribution curve showed that LAI did not significantly deviate from a normal distribution. Although it follows normal distribution, it is not a critical factor to consider when assessing the performance of nonparametric regression algorithms. The observed peanut LAI ranged from a minimum of 1.12 m^2^/m^2^ to a maximum of 7.5 m^2^/m^2^, with an average value of 3.79 m^2^/m^2^, indicating moderate to dense canopy (Figure 2). A study by Sarkar et al. [58] reported that the measured average peanut LAI in 2017 ranged from 0.8 to 2.6, and the values in 2019 varied from 1.5 to 5.8 with mean LAI values of 1.6 and 3.7 in 2017 and 2019, respectively. These values are consistent with the range of in situ mean peanut LAI values observed in the present study.

### 3.2. Assessment of Vegetation Indices for Estimation of Peanut LAI

**Figure 3:**
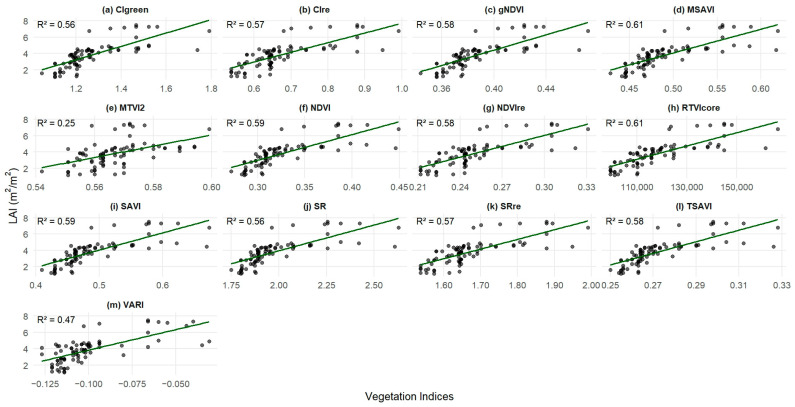
Relationship between peanut leaf area index and VIs: (**a**) CIgreen, (**b**) CIre, (**c**) gNDVI, (**d**) MSAVI, (**e**) MTVI2, (**f**) NDVI, (**g**) NDVIre, (**h**) RTVIcore, (**i**) SAVI, (**j**) SR, (**k**) SRre, (**l**) TSAVI, and (**m**) VARI.

All the PlanetScope-derived VIs showed sensitivity across the full range of peanut LAI values, from 1.12 m^2^/m^2^ to 7.5 m^2^/m^2^, with different data samples clustered around the best fit line and R^2^ ranging between 0.25 and 0.61 (Figure 3). Among the thirteen VIs, MSAVI and RTVIcore captured the LAI, with the highest coefficient of determination (R^2^ = 0.61), followed by NDVI and SAVI (R^2^ = 0.59). The findings show that NDVI and SAVI saturate at LAI values above 3 m^2^/m^2^, limiting their use for higher LAI, consistent with previous studies reporting strong correlations at low LAI but saturation at medium to high LAI [13]. Unlike NDVI and SAVI, which saturated at LAI values above 3 m^2^/m^2^, MSAVI and RTVIcore showed strong relationships with LAI without saturation across the full range of values (Figure 3d,h), consistent with previous studies on RTVIcore [12] and MSAVI [59]. These findings indicate that MSAVI and RTVIcore are suitable for monitoring peanut with dense canopies. In contrast, MTVI2 exhibited a weak relationship with LAI (R^2^ = 0.25); although it was strongly correlated with LAI in MERIS imagery [60], it proved less effective for peanut using PlanetScope, as many data points deviated from the 1:1 fit line.

### 3.3. Estimation of the Peanut LAI Based on Single Vegetation Indices

**Figure 4:**
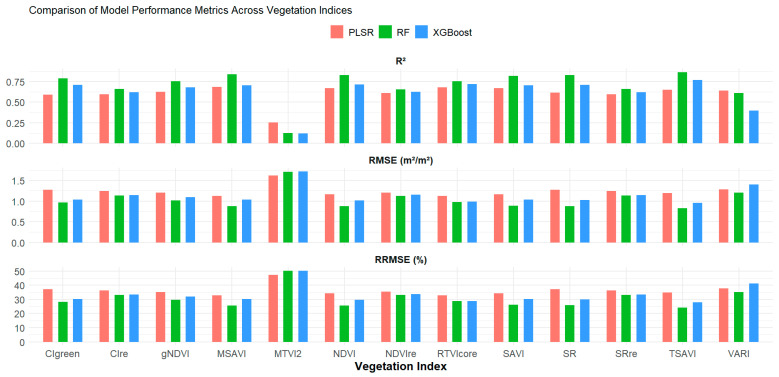
Validation metrics (R^2^, RMSE, and RRMSE) for peanut LAI estimation using single vegetation indices and regression models.

Three different regression models, namely RF, XGBoost, and PLSR, were used to estimate peanut LAI using single spectral vegetation indices (Table 1). Using individual vegetation indices, LAI estimation accuracy varied across models and indices, with RF generally outperforming XGBoost and PLSR (Figure 4). Across all indices, R^2^ ranged from 0.12 to 0.87 (RF), 0.12 to 0.77 (XGBoost), and 0.25 to 0.69 (PLSR); RMSE from 0.83 to 1.71 m^2^ m^−2^, 0.95 to 1.72 m^2^ m^−2^, and 1.12 to 1.61 m^2^ m^−2^; and RRMSE from 24.20 to 50.14%, 27.96 to 50.39%, and 32.88 to 47.36% for RF, XGBoost, and PLSR, respectively. Further results demonstrate that the TSAVI index provided the greatest accuracy for RF and XGBoost, respectively (RF: R^2^ = 0.87, RMSE = 0.83 m^2^/m^2^, RRMSE = 24.20%; XGBoost: R^2^ = 0.77, RMSE = 0.95 m^2^/m^2^, RRMSE = 27.96%). For the PLSR model, RTVIcore yielded the highest accuracy (R^2^ = 0.68, RMSE = 1.12 m^2^/m^2^, RRMSE = 32.88%). The results also showed poor agreement between measured and estimated LAI using the MTVI2 across all the models (Figure 4). This result contrasts with the study of Haboudane et al. [14], who reported that MTVI2 exhibited strong sensitivity to high LAI values greater than 4. However, the result from our study was consistent with the findings by Xie et al. [61], which demonstrated the poor performance of MTVI2 in estimating winter wheat LAI beyond 3 m^2^/m^2^. Our results suggest that the TSAVI (using the RF model) plays a significant role in the estimation of LAI. This result aligns with the results reported by [62], who showed that TSAVI provided the most accurate LAI estimates for LAI values below 4. TSAVI was developed to minimize soil brightness effects [36], and has been shown to be a useful index for estimating LAI by reducing soil influence in canopy reflectance models.

### 3.4. Selection of Important Variables for Peanut LAI Estimation

**Figure 5:**
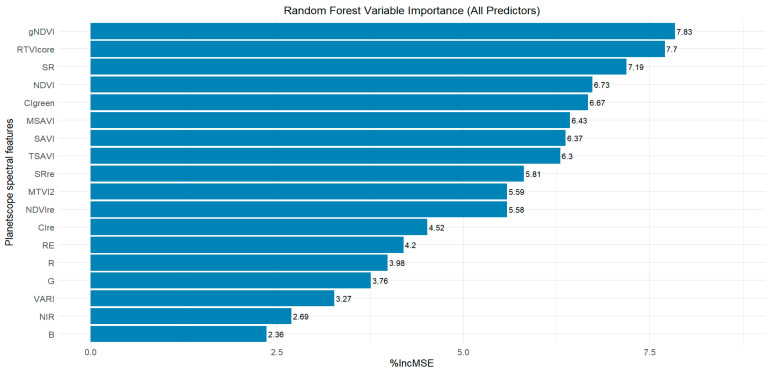
Feature importance plot generated by the RF algorithm from PlanetScope spectral data using the function *importance()* in the *randomForest* package. Higher %IncMSE values imply more impact on LAI estimation. The full names of spectral features (spectral bands and VIs) are presented in Table 1. RE, red-edge; SR, simple ratio; NDVI, normalized difference vegetation index; etc.

Variable screening plays a critical role in identifying the most influential input variables, thereby enhancing model estimation accuracy. This step is important, as evidenced in the literature, which underscores that input variables differ in their relevance and contribution to model performance, making the assessment of individual variable importance a fundamental aspect of the modeling process [63]. In this study, eighteen predictor variables (see Table 1) were evaluated for their relevant importance for LAI estimation. The results, as shown in Figure 5, revealed that for the PlanetScope spectral reflectances, the red-edge band was the most important predictor, followed by the red band, with %IncMSE values of 4.2 and 3.98, respectively, while the blue band was relatively the worst predictor with a %IncMSE of 2.36. Consistent with the findings of this study, Farmonov et al. [64] reported that the red-edge band 7 from PlanetScope imagery was the most influential spectral band for estimating wheat yield using the RF algorithm. Similarly, Kganyago et al. [21] demonstrated that the red-edge bands of Sentinel-2, particularly band 5 (705 nm), contributed most significantly to the performance of the RF algorithm for estimating LAI of multiple crops (i.e., maize, beans, and peanuts), which is similar to our findings. Reflectance in the red-edge spectral region is highly sensitive to vegetation conditions and has been recognized as a valuable source of information for agricultural monitoring [65].

Further results showed the varied impact of VIs on the accuracy for the RF model (Figure 5). Among the VIs, gNDVI was by far the most critical explanatory variable, followed by RTVIcore, with %IncMSE values of 7.83 and 7.7, respectively, while the VARI was the least important index (%IncMSE = 3.27). A study by Zhu et al. [66] demonstrated that VIs derived from the red-edge band, specifically SRre and NDVIre derived from WorldView-2 imagery, provided superior model accuracy for LAI estimation when employing the RF algorithm. However, in our study, the red-edge-based index (RTVIcore) derived from PlanetScope imagery was ranked as the second most important variable (after non-red-edge gNDVI) for estimating peanut LAI using the RF.

**Figure 6:**
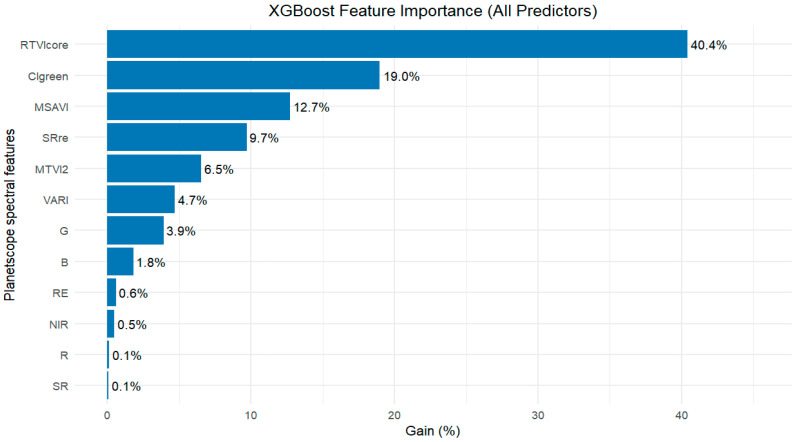
Feature importance plot generated by the XGBoost algorithm from PlanetScope spectral data, using the *xgb.importance()* function in *xgboost* package. Higher Gain (%) values represent more impact on model performance accuracy. Spectral features with 0% estimation power were excluded from the plot. The full names of spectral features (spectral bands and vegetation indices) are presented in Table 1. RE, red-edge; SR, simple ratio; etc.

Figure 6 shows that green contributed most to XGBoost model performance (Gain% = 3.9%), followed by the blue band (Gain% = 1.8%). In contrast, the red band had minimal importance (Gain% = 0.1%). A study by Liu et al. [48] reported that the NIR band (derived from multispectral unmanned aerial vehicle (UAV) data) yielded the highest accuracy in simulating rice LAI using both RF (R^2^ = 0.73, RMSE = 0.98) and XGBoost (R^2^ = 0.77, RMSE = 0.88) algorithms. In contrast, our findings indicate that the green band using XGBoost demonstrated greater performance for peanut LAI estimation. Further results showed that the RTVIcore index has the greatest influence on XGBoost model accuracy (i.e., Gain% = 40.4%), followed by CIgreen and MSAVI, with Gain% values of 19.0% and 12.7%, respectively, while SR showed poor influence on XGBoost performance (i.e., Gain% = 0.1%) (Figure 6).

**Figure 7:**
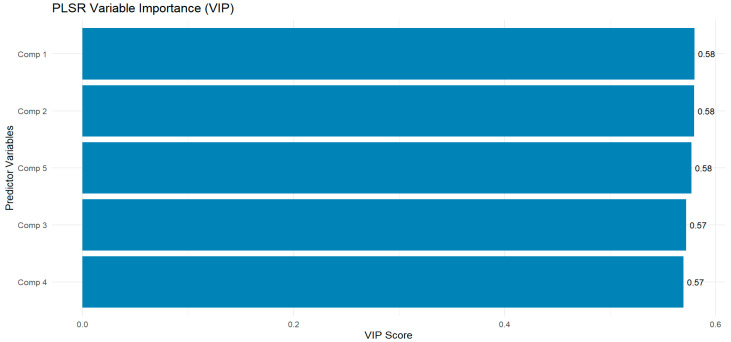
VIP scores of different components for estimating peanut LAI using the PLSR Variable importance in projection (VIP) function. Higher VIP score values are better indicators of how much each predictor influences LAI estimation. In this study, we selected predictors in PLS Comp 1.

**Figure 8:**
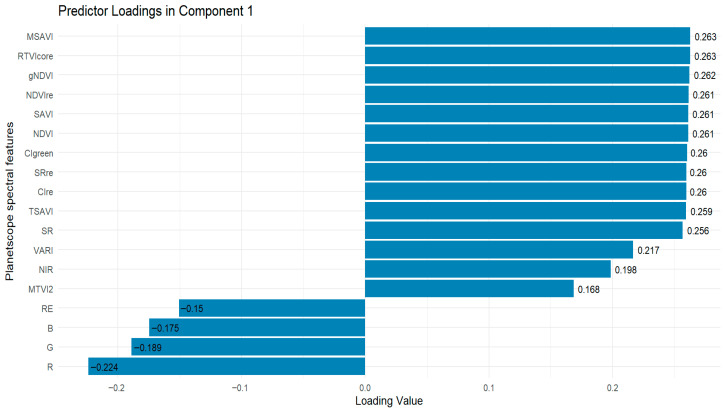
PLSR Variable importance in projection (VIP) function. Higher loading values indicate a stronger contribution of a variable to PLS Comp 1. In this study, the top six spectral features were selected for PLSR modeling.

For the PLSR, various PLS components were evaluated, and Component 1, identified as the most influential based on its VIP score of 0.58 (Figure 7) was selected for estimating LAI using the PLSR algorithm. The important spectral features that contributed to Comp 1’s influence on model performance indicate that the most important VIs were MSAVI and RTVIcore (loading values = 0.263), followed by gNDVI (loading value = 0.262), while the worst contributor was MTVI2 with the lowest loading value of 0.168 (Figure 8). All the spectral bands (red-edge, blue, green, and red), except the NIR band (loading value = 0.198), showed negative influence on model performance. This suggests that the inclusion of red-edge, blue, green, and red bands could reduce the accuracy of the PLSR algorithm for estimating peanut LAI.

### 3.5. Estimation of Peanut LAI Based on Machine Learning and Statistical Algorithms Using Multiple Predictor Variables

From the feature importance plots (Figure 5, Figure 6 and Figure 8), different data combinations of variables were used to train the RF, XGBoost, and PLSR algorithms, including the top three spectral bands, all spectral bands, the top six VIs, and all spectral features combined (i.e., spectral bands and VIs) (see Table 3).

We analyzed the spectral bands (the top three bands based on variable importance and all five spectral bands) as input variables in the three algorithms. The top three spectral bands based on feature importance for each algorithm are as follows: (red-edge, red, green), (green, blue, red-edge), and (red-edge, NIR, blue) for RF, XGBoost, and PLSR, respectively (Figure 5, Figure 6 and Figure 8). The five spectral bands used as predictors in all the algorithms include: red-edge, red, green, NIR, and blue. The results showed that incorporating all the spectral bands in the three algorithms as predictor variables led to improved LAI estimation accuracy compared to using only the top three bands based on feature importance (see Table 3).

Feature importance analysis of RF, XGBoost, and PLSR (Figure 5, Figure 6 and Figure 8) identified the top six VIs for peanut LAI estimation: RF—gNDVI, RTVIcore, SR, NDVI, CIgreen, MSAVI; XGBoost—RTVIcore, CIgreen, MSAVI, SRre, MTVI2, VARI; and PLSR—MSAVI, RTVIcore, gNDVI, NDVIre, SAVI, NDVI. Our results demonstrate that spectral VIs provide superior model accuracy in estimating LAI compared to the use of spectral bands alone (see Table 3). These findings are consistent with the results of Tunca et al. [67], who reported that using VIs computed from multiple spectral bands as input features were more effective for estimating sorghum LAI than using individual spectral bands alone. Similar findings were reported by Dube et al. [63], who demonstrated that integrating VIs with traditional red, green, blue, and near-infrared (RGBNIR) bands from RapidEye imagery improved LAI estimation accuracy by approximately 20% compared to using the RGBNIR bands or RGBNIR indices as independent predictor variables. In contrast to our findings, Chatterjee et al. [68] found that reflectance-based models outperformed vegetation index-based models, particularly during the mid to late vegetative stage of corn growth (by 5–15%) and at the silking stage (by 25%). According to Chatterjee et al. [68], the superior performance of reflectance-based models was primarily attributed to the red-edge and NIR bands, due to their increased sensitivity to higher LAI values.

We also tested the influence of using all the spectral features (spectral bands and VIs) on RF, XGBoost, and PLSR model performance accuracy. The findings underscore the critical role of feature selection in model calibration, as the results showed that incorporating all the spectral bands and VIs did not enhance the performance of the RF, XGBoost, and PLSR algorithms in estimating peanut LAI (see Table 3). For instance, in the case of the RF, incorporating all spectral features led to a reduction in model accuracy, with an RRMSE increase of 2.05% compared to using only the top six VIs. Similarly, for XGBoost, the use of all spectral features resulted in a slight RRMSE increase of 0.15%. The most significant reduction in accuracy was observed in the PLSR algorithm, where including all spectral features increased the RRMSE by 3.51% compared to using the top six VIs alone. These results are consistent with the results of Shen et al. [69], who demonstrated that using all the features in an algorithm does not necessarily improve estimation accuracy, and may result in reduced accuracy.

**Figure 9:**
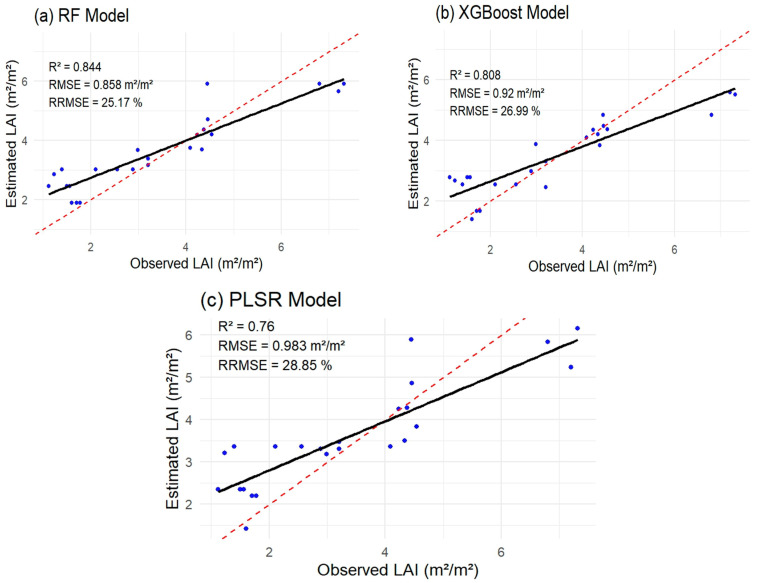
Scatterplots for leaf area index (LAI, m^2^/m^2^) showing the performance of (**a**) random forest (RF), (**b**) eXtreme Gradient Boosting (XGBoost), and (**c**) Partial Least Squares Regression (PLSR) with Planetscope data. The red dashed line represents the 1:1 line, indicating perfect agreement between observed and estimated LAI values. The solid black line is the regression (trend) line fitted to the data.

The LAI estimation results (Figure 9a–c) indicate that the RF achieved the highest estimation performance, with an RRMSE of 25.17% and an RMSE of 0.858 m^2^/m^2^, and explained 84.4% of LAI variability (Figure 9a). This was followed by the XGBoost algorithm, which yielded an RRMSE of 26.99%, an RMSE of 0.92 m^2^/m^2^, and an R^2^ of 0.808 (Figure 9b). The relatively similar RRMSE values of RF and XGBoost suggest that both algorithms exhibited comparable estimation capabilities in estimating peanut LAI. This is expected, as both methods are ensemble models based on decision trees [57], sharing fundamental characteristics such as the ability to reduce overfitting through ensemble strategies—bagging and boosting for RF and XGBoost, respectively. Moreover, ensemble tree-based models are inherently better suited to capturing nonlinear relationships between VIs and LAI [70]. In contrast, further results showed that the PLSR algorithm exhibited the least estimation accuracy, with an RRMSE of 28.85% and an RMSE of 0.983 m^2^/m^2^, and explained 76% of variability in LAI (Figure 9c). The findings of Intarat et al. [70] demonstrated that PLSR performed statistically worse in estimating rice LAI, which is consistent with our results. The relatively lower performance of the PLSR model in estimating LAI can be attributed to its limitation of dealing with nonlinear relationships between VIs and LAI [70,71].

Previous studies have shown the difficulty in using the traditional VIs to estimate crop LAI due to saturation of the VIs and their low sensitivity when medium to high LAI values are present [12,13]. In this study, however, we found the feasibility of estimating peanut LAI using multiple VIs derived from high-resolution PlanetScope imagery and nonparametric ML and statistical algorithms. Meanwhile, compared to using VI-based parametric regression models (using fitting functions such as linear, power, and exponential), nonparametric ML models are well-known for their ability to minimize multicollinearity issues [72,73]. While the RF and XGBoost algorithms both exhibited strong results compared to the PLSR in estimating peanut LAI, the RF algorithm demonstrated a slightly higher estimation accuracy, consistent with the findings reported in previous studies [21]. In contrast, Qiao et al. [19] demonstrated that the SVR model integrating vegetation indices and texture features from UAV imagery achieved superior accuracy in peanut LAI estimation (R^2^ = 0.867, RMSE = 0.491). Kganyago et al. [21] employed RF, sparse Partial Least Squares (sPLS), and Gradient Boosting Machine (GBM) to estimate LAI, leaf chlorophyll content (LCab), and canopy chlorophyll content (CCC) for maize, beans, and peanuts. Their results indicated that RF outperformed both sPLS and GBM across all three crops’ parameters, which is similar to our results. Recent studies have also demonstrated the superiority of RF methods in estimating LAI in various crops, such as in winter wheat crops [74], paddy rice [75], maize [76], winter rapeseed [77], and potato [78]. However, the superior performance of the RF algorithm in estimating peanut LAI in our study may be attributed to its robustness to noise and potential to capture nonlinear interactions among spectral input features. Therefore, integrating important VIs into the ML models, such as RF, can help mitigate the saturation effects commonly observed by using only single VIs.

**Figure 10:**
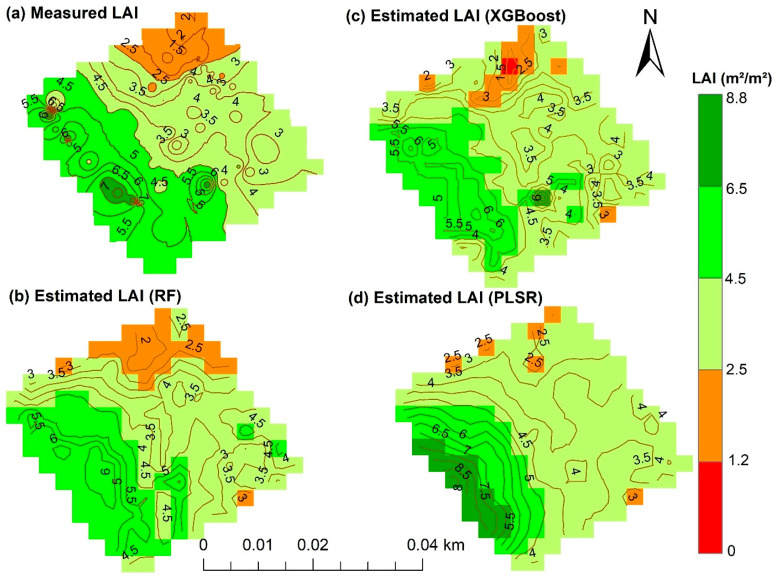
Maps of measured peanut LAI (**a**) and LAI estimates derived from RF (**b**), XGBoost (**c**), and PLSR (**d**).

The measured and estimated peanut LAI can be visually seen in Figure 10. The estimated LAI were obtained by RF, XGBoost, and PLSR models using the top six vegetation indices as shown in Figure 5, Figure 6 and Figure 8. The low and high LAI regions are depicted in red and dark green hues, respectively, which showed distinct variations in LAI within the field (Figure 10). As seen in the map, the RF model demonstrated robust performance, effectively capturing the variability of measured LAI (Figure 10b). The XGBoost-based estimation map showed a similar spatial pattern as the RF LAI map (Figure 10c). In contrast, PLSR showed a limited ability to capture the spatial variability of measured LAI, particularly overestimating LAI in areas with low observed values (Figure 10d), despite previous findings that PLSR can perform better for estimating lower values than other models [71]. The observed comparable and superior performance of RF and XGBoost in estimating peanut LAI in this study could be linked to the capability of these ensemble-based models to capture nonlinear relationships between LAI and spectral features derived from PlanetScope imagery, compared to PLSR, which is inherently limited in its ability to capture nonlinear interactions, thereby reducing its estimation performance.

## 4. Limitations and Recommendations

In this study, certain limitations should be admitted which would guide future studies. First, the in situ LAI dataset was relatively small, which may limit the robustness of model calibration and validation. Therefore, collecting more LAI samples would be required for accurate LAI estimation in future studies. Additionally, field measurements were conducted only at a single growth stage (i.e., stem elongation stage), limiting the evaluation of model performance across different peanut growth stages. Moreover, future studies should compare LAI estimations from the VitiCanopy app with those obtained using the LAI-2000 Plant Canopy Analyzer. Future studies will evaluate the transferability of RF-based LAI estimation models across different regions and environmental conditions to assess their robustness beyond the initial model training domain.

## 5. Conclusions

PlanetScope is a high-resolution optical Earth observation satellite constellation that provides near-daily global imagery, providing a reliable data source for estimating LAI for a wide range of crops. In this study, we investigated the performance of random forest (RF), eXtreme Gradient Boosting (XGBoost), and Partial Least Squares Regression (PLSR) algorithms in estimating peanut LAI using PlanetScope-derived spectral features (bands and VIs). Thirteen VIs were assessed for the estimation of peanut LAI. Overall, most VIs exhibited strong positive linear relationships with LAI, but showed some saturation when LAI reached 3 m^2^/m^2^. The thirteen VIs assessed individually revealed that their performance varied in estimating peanut LAI using RF, XGBoost, and PLSR models. The TSAVI index achieved the highest LAI estimation accuracy with RF (R^2^ = 0.87, RMSE = 0.83 m^2^ m^−2^, RRMSE = 24.20%) and XGBoost (R^2^ = 0.77, RMSE = 0.95 m^2^ m^−2^, RRMSE = 27.96%), while RTVIcore performed best with the PLSR model (R^2^ = 0.68, RMSE = 1.12 m^2^ m^−2^, RRMSE = 32.88%). Overall, the RF model using TSAVI achieved the highest accuracy for peanut LAI estimation among all single-index models.

Moreover, the spectral features (bands and VIs) that had the highest influence on the model accuracy were identified using the RF, XGBoost, and PLSR feature importance metrics. The gNDVI was identified as the most important predictor of peanut LAI using the RF model. The RTVIcore had the highest influence on XGBoost model performance accuracy. For the PLSR algorithm, the MSAVI and RTVIcore were the important predictors of LAI estimates. Further, our results demonstrated that spectral VIs exhibited greater accuracy compared to accuracy using spectral bands in the estimation of peanut LAI, with the RF model showing the overall best accuracy (R^2^ = 0.844, RMSE = 0.858 m^2^/m^2^, RRMSE = 25.17%). Meanwhile, the integration of spectral bands and VIs identified through variable importance analysis was evaluated for LAI estimation, and the results showed that combining bands and VIs led to a reduction in accuracy for LAI estimates, highlighting the significance of selecting important predictor variables to guarantee the best accuracy. Overall, our results demonstrated that the RF models provide the most robust performance for peanut LAI using PlanetScope-derived spectral VIs, thereby making it a strong candidate for operational applications in the context of peanut LAI monitoring.

## Figures and Tables

**Table 1:** PlanetScope spectral features used in this study.

Data	Predictor Variables	Description	Equation	References
PlanetScope multispectral surface reflectance	B	Blue (465–515 nm)	/	
	G	Green (547–583 nm)	/	
	R	Red (650–680 nm)	/	
	RE	Red-edge (697–713 nm)	/	
	NIR	Near-infrared (845–885 nm)	/	
PlanetScope vegetation indices	NDVI	Normalized difference vegetation index	$\left(R_{NIR}-R_{RED}\right)/\left(R_{NIR}+R_{RED}\right)$	[28]
	gNDVI	Green normalized difference vegetation index	$\left(R_{NIR}-R_{GREEN}\right)/\left(R_{NIR}+R_{GREEN}\right)$	[29]
	MSAVI	Modified soil-adjusted vegetation index	(2 × R_NIR_ + 1 − sqrt ((2 × R_NIR_ + 1)^2^ –8 × (R_NIR_ − R_RED_)))/2	[30]
	SAVI	Soil-adjusted vegetation index	(R_NIR_ − R_Red_)(1 + L)/(R_NIR_ + R_Red_ + L), where L = 0.5 represents soil adjustment factor.	[31]
	SR	Simple ratio	$R_{NIR}/R_{RED}$	[32]
	SR_RedEdge_	Red-edge simple ratio	$R_{NIR}/R_{RED-edge}$	[33]
	RTVI_core_	Red-edge triangular vegetation index (core only)	$100(R_{NIR}-R_{RED-edge})-10(R_{NIR}-R_{GREEN})$	[34]
	CI_Green_	Chlorophyll index-green	${(R}_{NIR}/R_{GREEN})-1$	[35]
	TSAVI	Transformed soil-adjusted vegetation index	a(R_NIR_ − aR_RED_ − b)/(R_RED_ + aR_NIR_ – a × b), where a = 0.33 and b = 0.5 are slope and intercept of a solid line, respectively, with an adjustment factor of 1.5	[36]
	MTVI2	Modified triangular vegetation index	${1.5\;[1.2(R}_{NIR}-R_{GREEN})-2.5(R_{RED}-R_{GREEN})]/\sqrt{\left[{\left(2R_{NIR}+1\right)}^{2}-(6R_{NIR}-5\sqrt{\left(RED\right)})-0.5\right]}$	[14]
	CI_RedEdge_	Red-edge chlorophyll index	${(R}_{NIR}/R_{RED-edge})-1\;$	[37]
	NDVI_RedEdge_	Red-edge normalized difference vegetation index	$\left(R_{NIR}-R_{RED-edge}\right)/\left(R_{NIR}+R_{RED-edge}\right)$	[33]
	VARI	Visible atmospherically resistant index	${(R}_{GREEN}-R_{RED})/{(R}_{GREEN}+R_{RED}-R_{BLUE})$	[38]

**Table 2:** Advantages and limitations of RF, XGBoost, and PLSR algorithms used in the study.

Algorithm	Model Parameters Used	Advantages	Limitations	References
Random Forest (RF)	ntree = 500, mtry = 3	Can provide interpretability and findings by analyzing both the main and alternative features within the decision trees.	It can be computationally demanding and slower in terms of processing speed, and offers lower interpretability compared to simpler regression models.	[42,56]
		Suitable for processing high-dimensional data and data with missing variables.		
Extreme Gradient Boosting (XGBoost)	eta = 0.1, max_depth = 6, nrounds = 500	Unaffected by highly correlated features, reduces the feature multicollinearity issues, and mitigates overfitting problems.	XGBoost training can be computationally intensive, and optimization to speed up computation is not always beneficial.	[46,57]
		Capability to handle sparse data, and nonlinearity integrating decision trees with boosting approaches.		
		Can overcome the limitations of computational speed and accuracy, requiring less training and estimation time.		
Partial Least Squares Regression (PLSR)	ncomp = 5	Efficiency in reducing dimensionality and ability to handle correlated features.	Limited interpretability of model coefficients.	[51]

ntree: number of decision trees; mtry: number of features at each split; eta: learning rate; ncomp: number of PLS components; max_depth: maximum depth of each decision tree; nrounds: number of boosting rounds.

**Table 3:** Validation statistics of PlanetScope spectral features for estimation of peanut LAI.

Input Variables	Algorithm	Number of Predictors	R^2^	RRMSE (%)	RMSE (m^2^/m^2^)
	RF	3	0.706	34.42	1.173
Top 3 spectral bands based on feature importance	XGBoost	3	0.733	28.40	0.968
	PLSR	3	0.657	33.71	1.149
	RF	5	0.793	30.27	1.032
All spectral bands	XGBoost	5	0.771	27.59	0.941
	PLSR	5	0.664	33.43	1.139
	RF	6	0.844	25.17	0.858
Top 6 VIs based on feature importance	XGBoost	6	0.808	26.99	0.92
	PLSR	6	0.76	28.85	0.983
	RF	13	0.791	27.22	0.928
All spectral features (Bands + VIs)	XGBoost	13	0.786	27.14	0.925
	PLSR	13	0.686	32.36	1.103

## Data Availability

The original contributions of this study are presented within the article. For additional information, researchers are encouraged to contact the corresponding author. The in situ peanut LAI data and raw R scripts used in the analysis are available upon request.
